# The Evolution, Current Landscape, and Future Prospects of Oncolytic Virotherapy in Melanoma: Talimogene Laherparepvec and Beyond

**DOI:** 10.3390/cells14201620

**Published:** 2025-10-17

**Authors:** John Smestad, John Rieth, Douglas Laux, Mohammed Milhem

**Affiliations:** 1 Department of Internal Medicine, University of Iowa Hospitals and Clinics, Iowa City, IA 52242, USA; 2 Physician-Scientist Training Program, University of Iowa Hospitals and Clinics, Iowa City, IA 52242, USA; 3 Holden Comprehensive Cancer Center, University of Iowa Health Care, Iowa City, IA 52242, USA

**Keywords:** talimogene laherparepvec, T-VEC, vusolimogene oderparepvec, RP1, oncolytic virus, melanoma, anti-tumor immunity

## Abstract

Oncolytic viruses represent an emerging class of therapeutic agents that have the potential to transform the care of patients with melanoma. In this narrative review, we describe the evolution of oncolytic virus approaches. We begin by describing early investigations using wild type viruses and then the development of sophisticated Herpes simplex virus 1 (HSV-1) variant constructs such as talimogene laherparepvec (T-VEC) and vusolimogene oderparepvec (Replimune-1, RP1), which incorporate deletions of viral genes and expression of human or synthetic transgenes to promote tumor selectivity, dendritic cell recruitment, antigen presentation, and stimulation of systemic anti-tumor immune responses. We review the status of clinical trials of oncolytic viruses in melanoma, highlight regulatory challenges, and describe important concepts and key remaining questions within the field. While T-VEC remains the only Food and Drug Administration (FDA)-approved oncolytic virus for melanoma treatment, ongoing research focusing on next-generation viral constructs and combination strategies aims to further improve clinical outcomes and expand the applicability of oncolytic virus therapy in melanoma.

## 1. A Brief History of Oncolytic Viruses

The field of modern oncolytic virus therapy traces its roots to early clinical observations dated to the late 1800s, noting the association of viral infections with temporary remissions in patients with hematologic malignancies [1,2,3,4]. The first therapeutic applications of viruses were attempted in cancer patients in the 1940s–1950s [5,6,7,8]. These early studies used agents such as wild type Hepatitis B virus, West Nile virus, Mumps virus, adenoidal pharyngeal conjunctival virus, Semliki Forest virus, and Newcastle Disease virus, with some suggestion of therapeutic responses, but overall poor safety profiles. More widespread evaluation of wild type viruses was hindered by inconsistent anti-tumor efficacy, lack of durable responses, unacceptable infectious toxicities including encephalitis, organ failure, and death, poor in vivo replication, and rapid neutralization and clearance by the host immune system [9,10].

**Figure 1:**
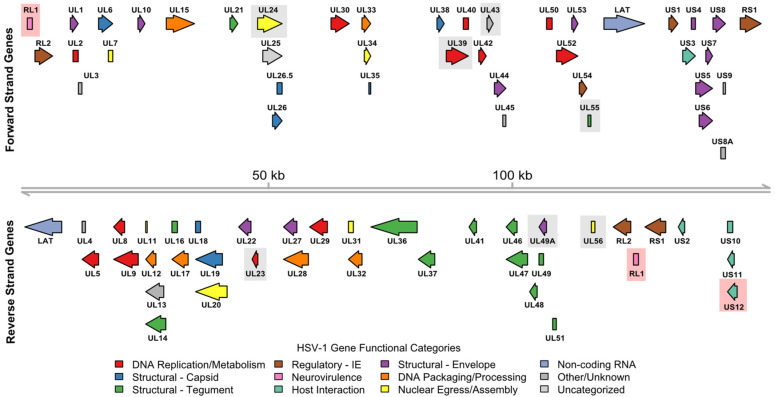
HSV-1 strain 17 viral genome open reading frame (ORF) functional annotations. The 152,222 bp linear duplex DNA HSV-1 viral genome is shown as a gray line with coordinates marked at 50 kb intervals. Genomic coordinates for 79 ORFs (77 protein-coding) were derived from the National Center for Biotechnology Information (NCBI) Reference Sequence Database (RefSeq) assembly GCF_000859985.2. Functional annotations for protein-coding ORFs were derived from UniProt and represented by arrow color. A legend describing the respective color mappings is also shown. For proteins with multiple functions, a single predominant functional annotation was selected at the discretion of the authors. Gray shaded boxes indicate specific gene deletions that have been tested in HSV-1 variant oncolytic viruses prior to development of T-VEC. Red shaded boxes indicate specific gene deletions present in T-VEC.

With the advent of genetic engineering technologies in the 1990s allowing for the manipulation of viral genomes, attempts to engineer multiple variant forms of wild type viruses have been reported, including adenoviruses ONYX-015 (containing E1B deletion) and DNX-2401 (containing E1A/E1B deletion), designed to lyse tumor cells deficient in p53, with early clinical trials of ONYX-015 showing an adequate safety profile but limited efficacy in multiple cancer types [11,12,13]. Herpes simplex virus type 1 (HSV-1) has become by far the most utilized viral backbone for oncolytic virus engineering due to the relative ease of manipulation of its large ~155 kbp duplex deoxyribonucleic acid (DNA) genome (Figure 1) [14], and natural cytolytic properties [9,10]. Early work to engineer HSV-1 for application in oncolytic viral therapy includes experiments published in 1991 by Martuza and colleagues demonstrating effective killing of human glioma cells and human glioma cells in subcutaneous cell line mouse xenograft and orthotopic xenograft models using a genetically engineered thymidine kinase-negative mutant Herpes simplex virus 1 (*dl*sptk) [15]. Although efficacious, the use of *dl*sptk was limited by fatal encephalitis at higher intracranial doses, prompting the design of additional HSV-1 mutants with further attenuation of neurovirulence capable of eliminating intracranial gliomas in an orthotopic mouse model [16].

Subsequent efforts to tailor HSV-1 for oncolytic therapy include modifications to restrict viral replication to tumor cells, reduce neurovirulence, and enhance immunogenicity. For clarity, in addition to describing these modifications below, we summarize the major genome modifications and clinical trial results for the many HSV-1 variant viruses that advanced to human testing in Table 1, and annotate these modifications onto the HSV-1 strain 17 reference genome shown in Figure 1.

Work by Minta et al. published in 1995 described the G207 HSV-1 mutant with insertional mutation in the UL39 gene (encoding ICP6 viral ribonucleotide reductase subunit 1) and deletion of both copies of the RL1 (γ34.5) gene (encoding ICP34.5, which functions in blocking cellular stress response to virus), both entities being crucial for viral replication and neurovirulence [17]. Following further preclinical testing [18,19], the G207 virus was eventually tested in a phase Ib clinical trial of recurrent glioblastoma (GBM), with an acceptable safety profile but underwhelming efficacy signal [20]. NV1020 is another HSV-1 mutant that was engineered with deletion of a single copy of the RL1 (γ34.5) gene, both copies of the UL56 gene (which encodes a viral protein that interacts with host cGAS protein, inhibiting host innate immune response), and deletion of UL24 (required for efficient viral replication) [21,22,23]. The UL56 protein is also involved in regulating cell-to-cell spread of the virus and is found within the virion envelope. In a phase I/II clinical trial of NV1020 given via hepatic artery infusion to patients with colorectal cancer hepatic metastases, treatment was relatively well-tolerated, with some partial responses observed [24,25,26]. HSV1716, an avirulent but replication-competent HSV-1 variant bearing a spontaneous deletion of a 759 bp fragment of the RL1 (γ34.5) gene, was tested in a phase I trial of five patients with advanced cutaneous melanoma, demonstrating safety of intralesional administration as well as flattening of melanoma nodules in patients receiving multiple injections [27]. Another variant form of HSV-1, HF10, was generated via spontaneous deletion of genomic regions containing multiple viral genes including UL43 envelope protein, UL49.5 envelope glycoprotein N, UL55 nuclear protein, UL56 membrane protein, US11 tegument protein, and US12 TAP transport inhibitor ICP47. It was tested in multiple clinical trials with evidence of modest efficacy, including a phase II clinical trial of HF10 given in combination with ipilimumab in cutaneous and mucosal melanoma [28].

**Figure 2:**
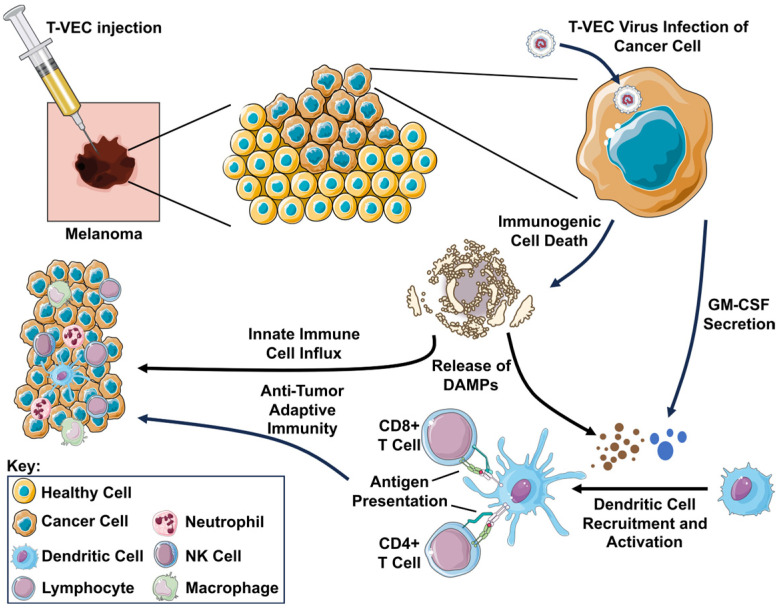
Schematic representation of T-VEC mechanism of action. Following injection, the T-VEC virus infects the cancer cell. The infected cell secretes GM-CSF (represented as blue circles) encoded as transgene in the virus, recruiting and activating dendritic cells to present tumor antigens to CD8+ T cells via MHC class I molecules and CD4+ T cells via MHC class II molecules, resulting in systemic anti-tumor immunity. Viral replication in the tumor cell results in immunogenic cell death, releasing damage-associated molecular patterns (DAMPs; represented as brown circles) including surface-exposed calreticulin, adenosine triphosphate (ATP), and high mobility group B1 (HMGB1), further promoting the influx of innate immune cells and antigen-presenting cells into the tumor. For further information on any abbreviations used in this figure, please reference the list of abbreviations provided elsewhere in this manuscript.

The most well-known among the HSV-1-based oncolytic viruses is talimogene laherparepvec (T-VEC), initially reported by Liu et al. in 2003, which has gone on to become the first oncolytic virus approved for clinical use in cutaneous melanoma [29]. Key design elements of T-VEC include deletion of both copies of RL1 encoding ICP34.5 (designed to restrict viral replication to tumor cells with defective interferon response), deletion of US12 encoding ICP47 (preventing viral suppression of major histocompatibility complex (MHC) class I molecules, resulting in more effective antigen presentation), and insertion of human granulocyte–monocyte colony-stimulating factor (GM-CSF; promoting dendritic cell recruitment with goal of augmenting systemic anti-tumor immunity). Tissue culture studies demonstrated that T-VEC selectively induces lysis of melanoma cells, with minimal impact on normal cells [29]. Testing of T-VEC efficacy in mouse syngeneic cell line xenograft models using T-VEC or murine analog OncoVEX^mGM-CSF^ (expressing murine GM-CSF) resulted in robust shrinkage of tumors, including distant lesions, suggestive of an abscopal effect [30]. Mechanistic studies demonstrated that, in addition to potent direct cell lysis, OncoVEX^mGM-CSF^ induces immunogenic cell death and potent anti-tumor immunity, with additional demonstration that adoptive transfer of cluster of differentiation 8 (CD8)+ T cells from cured mice confers protection against tumor challenge [31]. Importantly, the potent anti-tumor immunity promoted by T-VEC was further demonstrated to exhibit oncolytic properties and abscopal response in a murine cell line xenograft model with low stimulator of interferon genes (STING) expression, associated with resistance to anti-programmed cell death protein 1 (PD-1) immune checkpoint blockade [32]. Multiple studies have additionally demonstrated synergy of combining OncoVEX^mGM-CSF^ with anti-PD-1 and/or anti-Cytotoxic T-Lymphocyte-Associated Protein 4 (CTLA-4) blockade [30,31,33]. We present in Figure 2 a visual representation summarizing the known mechanisms of T-VEC action.

## 2. Oncolytic Virus Clinical Trials in Advanced or Metastatic Melanoma

The OPTiM trial was a multinational, randomized, open-label phase III study evaluating intratumoral T-VEC versus subcutaneous GM-CSF in patients with unresectable stage IIIB-IV cutaneous melanoma (Table 2) [34,35]. The trial enrolled 436 subjects in the United States, the United Kingdom, Canada, and South Africa, randomized 2:1 to receive T-VEC or GM-CSF for up to 24 months. T-VEC was injected into cutaneous/subcutaneous or nodal lesions ≥ 10 mm, given initially at a concentration of 10^6^ pfu/mL, with subsequent doses at 10^8^ pfu/mL, and injection volumes ranging from 0.1 mL for lesions ≤ 0.5 cm to 4.0 mL for lesions ≥ 5 cm, with total injection volume per visit capped at 4.0 mL, given every 2 weeks. GM-CSF was given subcutaneously at 125 µg/m^2^ daily for 2 weeks in a 4-week cycle. This trial demonstrated improvement in durable response rate (DRR), defined as a continuous response lasting ≥6 months, in the T-VEC arm compared to GM-CSF (16.3% vs. 2.1%, unadjusted odds ratio (OR), 8.9; 95% confidence interval (CI), 2.7 to 29.2; *p* < 0.001). The trial additionally demonstrated improved objective response rate (ORR: 31.5% with T-VEC and 6.4% with GM-CSF; descriptive *p* < 0.0001). The primary trial analysis did not show improvement in overall survival (OS), with median OS of 23.3 months with T-VEC and 18.9 months with GM-CSF (hazard ratio (HR), 0.79; 95% CI, 0.62 to 1.00; *p* = 0.051) [35]. Of note, a final planned descriptive analysis of the trial later on did suggest an overall survival benefit (OS: T-VEC median OS of 23.3 months compared to 18.9 months with GM-CSF; descriptive *p* = 0.0494) [34]. Treatment-related adverse events (TRAEs) grade ≥ 3 were observed in 11.3% of patients (most commonly cellulitis), with no grade 5 events. Based on these data, T-VEC was approved by the Food and Drug Administration (FDA) for the treatment of unresectable melanoma with cutaneous, subcutaneous, or nodal involvement on 27 October 2015, becoming a first-in-class approved therapeutic for melanoma [36].

Multiple clinical trials have subsequently investigated whether the addition of an immune checkpoint inhibitor (ICI) to T-VEC improves outcomes for patients with advanced melanoma, predicated upon substantial preclinical data suggesting the synergistic benefit of combining these therapeutic classes [30,31,33]. MASTERKEY-265 was a multicenter phase Ib/III trial investigating whether the addition of T-VEC to pembrolizumab improves outcomes for patients with stage IIIB-IV cutaneous melanoma. The results of the phase Ib portion of the study were very encouraging, with an overall response rate (ORR) of 62% and a complete response rate (CRR) of 43% in the 21 patients studied [37]. The phase III portion of the study was conducted as a multicenter, placebo-controlled, double-blinded, randomized design (Table 2) [38]. Of note, patients with prior anti-PD-1 or anti-Programmed Death Ligand 1 (PD-L1) ICI were excluded from enrollment. The trial enrolled 692 patients who were randomized in a 1:1 fashion. T-VEC was injected into accessible visceral or soft tissue ≥ 10 mm, or nodal lesions ≥ 15 mm on short axis. T-VEC was given at an initial concentration of 10^6^ pfu/mL, with subsequent doses at 10^8^ pfu/mL, and injection volume capped at 4 mL, given every 2 weeks until week 9, at which time, patients were transitioned to injections every 3 weeks. The trial did not meet its co-primary endpoints of progression-free survival (PFS) and OS improvement over Pembrolizumab monotherapy. Of note, the ORR observed in phase III was much lower than in phase IB (48% vs. 62%). The reason for this remains unclear. The trial noted similar frequency of grade ≥ 3 adverse events between treatment and control arms, without suggestion of overlapping toxicities.

Another open-label, multicenter, randomized phase II trial enrolled 198 patients to evaluate whether addition of T-VEC to ipilimumab 3 mg/kg improves clinical outcomes for patients with stage IIIB-IV cutaneous melanoma (Table 2) [39]. Inclusion criteria included measurable disease, defined as at least one lesion ≥ 10 mm for visceral or soft tissue disease, lymph nodes ≥ 15 mm in short axis, or cutaneous/subcutaneous lesions with short axis ≥ 5 mm. Injectable lesions were defined as cutaneous, subcutaneous, or nodal melanoma ≥ 5 mm in longest diameter. T-VEC dosing was similar to the OPTiM trial; ipilimumab 3 mg/kg was given every 3 weeks for up to 4 doses. The trial met its primary endpoint of investigator-assessed ORR (35.7% versus 16.0%), also showing higher DRR (33.7% vs. 13.0%); however, no PFS or OS benefit at 5 years was observed. High rates of grade ≥ 3 adverse events were noted in both arms of the trial (46.3% in treatment arm versus 43.2% in control arm), consistent with the known toxicities of high-dose ipilimumab. To date, no clinical trial has yet demonstrated a survival benefit of adding an immune checkpoint inhibitor to oncolytic virus therapy for melanoma, despite the promising preclinical data and theoretical synergy between these two therapeutic classes.

Given the fact that anti-PD-1/PD-L1 therapy has now become the backbone of systemic therapy for advanced or metastatic melanoma, a clinically relevant question is whether oncolytic virus therapy offers a benefit following progression on prior anti-PD-1 therapy. IGNYTE was a single-arm open-label trial testing the efficacy of adding of vusolimogene oderparepvec (RP1), a next-generation HSV-1-modified virus containing deletions of two copies of RL1 encoding ICP34.5, deletion of US12 encoding ICP47, engineered up-regulation of US11 via placing under control of a strong constitutively active promoter, transgenic expression of human GM-CSF, and transgenic expression of Gibbon ape leukemia virus fusogenic glycoprotein (GALV-GP R-), to nivolumab in patients with anti-PD-1-failed melanoma [40]. Features of the RP1 virus that represent improvements over T-VEC include the engineered overexpression of US11 designed to enhance replication within tumors having a defective antiviral response, and transgenic expression of GALV-GP-R- designed to enhance direct tumor killing via syncytia formation. Of note, 46.4% of patients enrolled in the IGNYTE trial had received prior dual anti-PD-1 and anti-CTLA-4 therapy, a subset of patients with generally very poor prognosis and few remaining treatment options. ORR in the enrolled population was 32.9%, including 15% with complete response (CR), with responses occurring in both injected and non-injected lesions with similar frequency (Table 2). This compares favorably to historical data on response rates for patients with continued anti-PD-1 therapy beyond progression (estimated ~6%) [41]. The median duration of responses to RP1 was 33.7 months, which also compares quite favorably with prior published data on T-VEC data from the OPTiM trial, showing that 19% of responses last longer than 6 months. The treatment was well-tolerated, with 12.9% of patients having grade ≥ 3 TRAEs, and no grade 5 events. The regulatory status of RP1 remains unsettled, however, as despite demonstrating clinically meaningful and durable responses in anti-PD-1-failed melanoma in the IGNYTE trial, the FDA has withheld approval pending confirmatory randomized data showing overall survival benefit, highlighting the ongoing debate over the sufficiency of single-arm response-based evidence for new therapies in this high-need population. This controversy may be resolved by the confirmatory IGNYTE-3 trial which is an ongoing randomized phase III trial evaluating RP1 plus nivolumab versus standard-of-care in patients with advanced melanoma who have progressed after prior anti-PD-1 and anti-CTLA-4 therapy [42].

Multiple other next-generation oncolytic viruses have additionally entered early phase clinical trials for advanced/metastatic melanoma. OH2 is a next-generation oncolytic virus derived from an HSV-2 backbone with potentially higher tumor selectivity compared to existing HSV-1-derived constructs, which was tested in a phase Ia/Ib trial enrolling 44 patients with stage III/IV melanoma of a primarily acral subtype in a Chinese population [43]. ORR was 37%, with durable responses ≥6 months observed in 29.6% of patients, and no TRAEs grade ≥ 3. Of note, 7 of 12 patients (58%) who had previously received anti-PD-1 therapy responded to OH2. A phase III confirmatory trial is ongoing in China. OrienX010 is another HSV-1-derived oncolytic virus that is similar to T-VEC with deletions of both ICP34.5 and ICP47 and over-expression of human GM-CSF. A phase Ib trial of OrienX010 in Chinese patients with stage IIIC-IV melanoma (predominantly acral and mucosal subtypes) demonstrated an ORR of 19.2% and a disease-control rate (DCR) of 54.6%, with responses observed in both injected and non-injected lesions [44]. Of note, the trial demonstrated an acceptable safety profile for injections up to 10 mL of 8E7 pfu/mL. A second phase Ib clinical trial in China investigated the use of OrienX010 in combination with Toripalimab (anti-PD-1 mAb) in patients with stage IV melanoma with injectable liver metastases [45]. The trial enrolled fifteen patients (60% mucosal, 20% cutaneous, 13.3% unknown, 6.7% acral) who received US-guided injection of hepatic lesions OrienX010 Q2W (8E7 pfu/mL, 10 mL per injection) until intolerance or disease progression, with repeat biopsy at 8–12 weeks following treatment initiation. ORR was 13.3% and DCR was 46.7%, with responses observed in both injected and non-injected lesions. Repeat biopsy at 8–12 weeks showed no residual melanoma cells in 30% of patients: two with partial response (PR), three with stable disease (SD). In total, 46.7% of lesions were noted to have impressive T cell infiltration compared to baseline.

“Next-generation” oncolytic viruses have been engineered to express additional molecules to further engage immune checkpoints and T cell receptors, with the goal of achieving enhanced anti-tumor immunity [46]. Replimune-2 (RP2) is a modified HSV-1 virus similar to RP1, but with the addition of anti-CTLA-4 antibody-like molecules designed to locally block CTLA-4 within tumor microenvironment. An open-label phase I study is currently in process investigating the safety and preliminary efficacy of RP2 given with or without nivolumab in patients with advanced solid tumors, including uveal melanoma [47]. A preliminary report on the efficacy of RP2 given with or without ICI in 17 patients with metastatic uveal melanoma who had progressed on or could not tolerate standard therapy showed an ORR of 29.4% (all partial responses) and was otherwise well-tolerated [48]. RP-202 is a currently recruiting randomized phase II/III trial in patients with metastatic uveal melanoma that will investigate the effectiveness of RP2 with nivolumab compared to ipilimumab with nivolumab in patients without prior treatment with immune checkpoint inhibitor [49]. BS006 is another HSV-2-based engineered oncolytic virus designed to express a PD-L1/CD3 bispecific antibody-like molecule within a tumor, currently undergoing phase I testing in melanoma and other advanced solid tumors [46,50].

## 3. Neoadjuvant Oncolytic Virus Trials in Melanoma

Multiple clinical trials have investigated the application of oncolytic viruses in melanoma in the neoadjuvant setting. A multicenter, open-label, randomized phase II trial previously investigated the use of T-VEC in the neoadjuvant setting for stage IIIB to IVM1a melanoma in patients with at least one injectable cutaneous, subcutaneous, or nodal lesion. The trial enrolled 150 patients from 48 sites in nine countries, randomizing 1:1 to receive six doses of neoadjuvant T-VEC or immediate surgery. T-VEC was given at an initial concentration of 10^6^ pfu/mL, with subsequent doses at 10^8^ pfu/mL, and injection volume capped at 4 mL, given every 2 weeks until surgery, no further injectable lesions, or intolerance. Additional adjuvant therapies were allowed at the investigator’s discretion. The primary endpoint for the trial was recurrence-free survival (RFS), with secondary endpoints including OS, local RFS, regional RFS, event-free survival (EFS), and distant metastasis-free survival (DMFS). At 5 years, the T-VEC group was reported to have superior RFS, EFS, DMFS, and OS compared to standard surgery (Table 3) [51]. Neoadjuvant use of T-VEC in cutaneous melanoma remains investigational, however, as neither arm in this trial included an immune checkpoint inhibitor, which would now be standard-of-care. Potential for the application of T-VEC or similar OVs in the neoadjuvant setting remains high, but additional trials are needed to investigate efficacy when given with current standard-of-care regimens including immune checkpoint inhibitors. NIVEC is an ongoing phase II single-arm trial evaluating T-VEC given in combination with nivolumab in resectable stage IIIB-IVM1a melanoma with injectable disease. The trial enrolled 24 patients who received four doses of T-VEC 4 mL with nivolumab every 2 weeks prior to surgery. Early results show a major pathologic response rate of 65% and 1-year EFS of 75%, with acceptable safety profile (Table 3) [52].

Additional neoadjuvant trials include a Phase Ib trial examining the use of OrienX010 given with Toripalimab in resectable acral melanoma given for 12 weeks prior to surgery [53]. In this study, OrienX010 was given at a maximum dose of 8E8 pfu up to 10 mL total volume Q2 weeks, with Toripalimab given 3 mg/kg Q3 weeks for up to 1 year. Pathological response rate was reported to be 77.8% (14.8% CR), with 2-year recurrence-free survival (RFS) of 81.5%. Acral melanoma is recognized as a rare and aggressive melanoma subtype with lower response to immunotherapy compared with other subtypes of cutaneous melanoma. Absence of robust data on the pathologic response rate of ICI alone in acral melanoma make the data from this trial difficult to interpret, as it is unclear whether OrienX010 provides benefit beyond that offered by ICI alone. KEYMAKER-U02 is an ongoing phase I/II trial investigating the neoadjuvant use of pembrolizumab given in combination with other agents in resectable stage IIIB-D melanoma [54]. One of the trial arms includes Pembrolizumab given with gebasaxturev (coxsackievirus A21). Preliminary results do not seem to show a strong efficacy signal as the pathologic response rate of Pembrolizumab plus gebasaxturev is not higher than Pembrolizumab monotherapy (pCR rate 40% vs. 47%, respectively). Long-term follow-up data from this trial are still maturing. At this time, the use of oncolytic viruses in the neoadjuvant setting remains investigational; outside of a clinical trial setting, the current standard-of-care for resectable disease stage IIIB-IVM1a remains neoadjuvant anti-PD-1 therapy.

## 4. Discussion

### 4.1. Evolution in Design of Oncolytic Viruses for Melanoma Therapy

HSV-1-derived oncolytic virus variants have been the most clinically studied and successful viruses to enter melanoma clinical trials. T-VEC, OrienX010, and many subsequent “next-generation” oncolytic viruses share specific modifications to the HSV-1 backbone, including deletion of ICP34.5 to attenuate neurovirulence and restrict viral replication to tumor cells, deletion of ICP47 to enhance immunogenicity, and engineered expression of human GM-CSF to stimulate dendritic cell response and prime the adaptive immune system for systemic anti-tumor T cell response [34,37,38,39,44,45,51,53]. These engineered elements result in direct tumor cell lysis caused by viral replication, releasing tumor antigens and resulting in local inflammation and the release of immunogenic cell death signals that synergize with GM-CSF to activate antigen presentation via dendritic cells, resulting in enhanced adaptive anti-tumor immunity with responses in even non-injected lesions via the abscopal effect [30,31,33].

Many “next-generation” oncolytic viruses show potential beyond that of T-VEC due to the incorporation of additional engineered elements to augment anti-tumor efficacy [46]. Beyond the core design elements of T-VEC, RP1 additionally includes engineered up-regulation of US11 via placing under control of a strong constitutively active promoter leading to enhanced tumor-specific replication, and transgenic expression of GALV-GP R-, which is a truncated envelope glycoprotein from gibbon ape leukemia virus that enhances cell-to-cell fusion, promoting direct lysis of tumor cells and enhancing the spread of the virus within tumors. RP2 retains the design features of RP1, with the added expression of an anti-CTLA-4 antibody-like molecule designed to be expressed within tumors and secreted into the local tumor microenvironment for local CTLA-4 blockade on T cells within tumors [55]. BS006 represents a departure from HSV-1-derived OVs, instead using HSV-2 due to superior oncolytic activity and cell-to-cell spread within tumors, especially in tumors that express the nectin-2 receptor [50]. This virus includes the addition of engineered expression of a PD-L1/CD3 bispecific antibody-like molecule that is secreted by tumors, designed to create an artificial immune synapse between CD3+ T cells and PD-L1-expressing tumor cells. The approach of expressing immune checkpoint inhibitor molecules directly within tumors, exemplified by RP2 and BS006, remains a very promising strategy.

### 4.2. Current Status of Clinical Application of Oncolytic Viruses in Melanoma Therapy

#### 4.2.1. Limited Scope of Current T-VEC Approval

**Figure 3:**
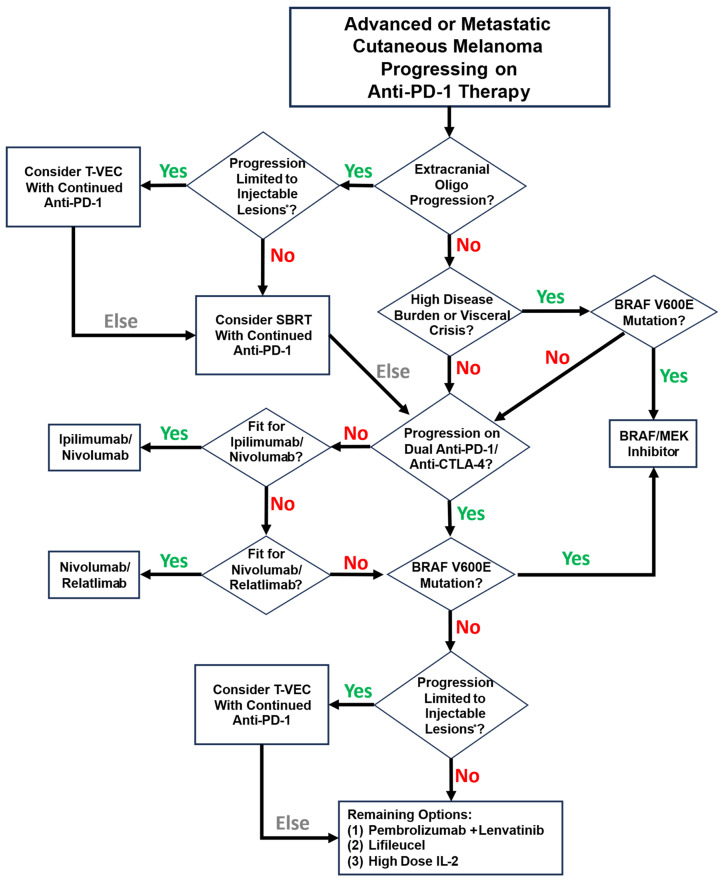
A reasonable approach to systemic therapy for patients with advanced or metastatic cutaneous melanoma progressing on anti-PD-1 therapy. Note that this schema reflects individual practice preferences based on clinical experience. * Injectable lesions are defined by the Imlygic package insert as “cutaneous, subcutaneous, and/or nodal lesions that are visible, palpable, or detectable by ultrasound guidance”.

Based on the phase III OPTiM trial demonstrating an ORR of 31.5% with DRR 19% of T-VEC in patients with unresectable stage IIIB-IVM1c melanoma, superior to effects of subcutaneous GM-CSF, T-VEC remains the only oncolytic virus FDA-approved for use in melanoma [34]. Trial data demonstrated ORR 31.5%, with 19% of responses durable beyond 6 months. The overall survival benefit of T-VEC in advanced melanoma has not been demonstrated. The current T-VEC approval is limited to the local treatment of unresectable cutaneous, subcutaneous, and nodal lesions in patients with melanoma recurrent after initial surgery [36]. Key populations excluded from the OPTiM trial include patients with non-cutaneous melanoma subtypes, patients with bone or CNS metastases, >3 visceral metastases, any visceral metastases >3 cm, patients with enlarging liver lesions, patients with LDH > 1.5x ULN, and patients with ECOG > 1. In practice, many patients with advanced melanoma fall outside of these criteria; thus, the applicability of the technology remains limited to very specific situations. To illustrate this, we present in Figure 3 a summary of a reasonable approach to the treatment of advanced or metastatic cutaneous melanoma progressing on anti-PD-1 therapy.

#### 4.2.2. Emerging Regulatory Challenges Confounding Clinical Translation

The IGNYTE single-arm trial investigated the use of RP1 oncolytic virus in patients with PD-1-failed melanoma, demonstrating an ORR of 32.9% (including 15% CR), with a median duration of response of 33.7 months, comparing favorably to prior T-VEC data. Notably, response rates in injected visceral lesions appear to be much higher with RP1 than with T-VEC (RP1 ORR of 41.7% to injected visceral lesions, compared to 15% with T-VEC). Despite the promising efficacy and durability of responses observed in the IGNYTE trial, the United States Food and Drug Administration (FDA) has rejected the drug approval application for RP1, resulting in uncertainty about the future of this agent [56]. This decision has generated confusion and concern within the oncology community, particularly regarding the regulatory pathway for drugs evaluated in single-arm trials for high-need, rare patient populations. For patients with advanced melanoma that has progressed on immune checkpoint inhibitor therapy, few options remain. The lack of availability of RP1 outside of clinical trials effectively limits clinicians to the use of T-VEC, which is generally regarded as a less potent oncolytic virus. We anticipate that the confirmatory randomized IGNYTE-3 trial will be slow to accrue given its very restrictive inclusion criteria [42].

### 4.3. Open Questions Surrounding the Use of Oncolytic Viruses in Melanoma

#### 4.3.1. Do OVs Have a Role in the Neoadjuvant Setting in the ICI Era?

Beyond the use of oncolytic viruses in the setting of advanced disease, T-VEC use in the neoadjuvant setting has shown a promising efficacy signal, but applicability remains unclear given the lack of concomitant administration of standard-of-care immune checkpoint inhibitor regimens [51]. The NIVEC trial is an ongoing phase II single-arm study evaluating T-VEC given in combination with nivolumab in patients with stage IIIB-IVM1a melanoma, with encouraging early pathological response data [52]. Survival data from this trial are yet to mature. Early phase clinical trial data from China have additionally demonstrated promising results for OrienX010 given in combination with Toripalimab in resectable acral melanoma, with pathological response rates reported at 77.8%- and a 2-year RFS of 81.5%, although the lack of clear data on response rates with Toripalimab alone makes the interpretation of these data challenging [53].

#### 4.3.2. What Dose of T-VEC Is Both Safe and Maximally Effective?

The current FDA-approved dose for T-VEC in advanced melanoma is capped at 4 mL of 10^8^ pfu/mL per administration [36]. The rationale for limiting T-VEC dose has been to avoid the devastating impacts of disseminated herpesvirus infections, which have been reported in immunocompromised individuals [57]. For this reason, T-VEC remains contraindicated in immunocompromised individuals [36], even as the ongoing phase Ib/II ARTACUS trial investigates the safety of RP1 in immune-suppressed solid organ transplant patients [58]. Initial results from the ARTACUS trial have not shown any new safety signals, suggesting that OVs may be safe to use in certain immune-suppressed populations [59]. Even in immunocompetent individuals, a theoretical risk exists that T-VEC can lead to herpetic infections at non-injected sites. In one widely cited study of T-VEC biodistribution and shedding, T-VEC DNA was detected by PCR from suspected herpetic mucosal and skin lesions in three individuals, in addition to the more ubiquitous pattern of isolation of T-VEC DNA from blood and urine of most study subjects, although the suspected herpetic lesions and body fluids were not demonstrated to be infectious in a cell culture assay [60]. To date, disseminated life-threatening infections have not been reported following T-VEC administration in immunocompetent individuals, and it remains unclear if patients would incur significant additional risk at higher doses. A higher T-VEC dose of up to 8 mL at concentration of 10^8^ pfu/mL given every 3 weeks was used in a reported phase Ib/III study in relapsed or metastatic head and neck squamous cell carcinoma [61]. A total of 36 patients were enrolled. The study noted one treatment-related grade 5 event due to arterial hemorrhage following T-VEC administration in a patient with a tumor encasing the right carotid artery, with an otherwise low (13.9%) risk of TRAEs grade ≥ 3. These safety data suggest that 8 mL dose of T-VEC may be safe to explore in other disease areas, including melanoma. Of note, the 8 mL dose of T-VEC is being investigated in conjunction with preoperative radiation in an ongoing trial for patients with soft tissue sarcomas [62].

#### 4.3.3. How Should Oncolytic Virus Dose Scale with Respect to Tumor Size and Disease Burden?

**Figure 4:**
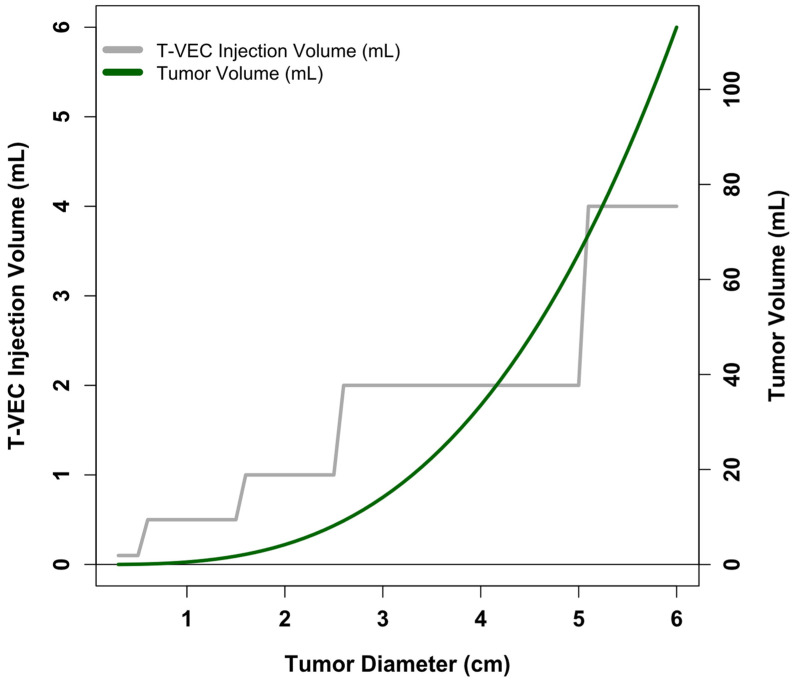
T-VEC dosing levels and calculated tumor volume as a function of tumor diameter. X-axis shows tumor diameter. Gray line and corresponding left-sided y-axis show the maximum T-VEC injection volume as a function of tumor diameter. Information derived from the Imlygic package insert. Green line and corresponding right-sided y-axis show calculated tumor volume as a function of tumor diameter, assuming spherical tumors ($V=\frac{\pi }{6}d^{3}$).

Beyond simply increasing the upper limit for injectable T-VEC dose, there is also opportunity for improvement within the field of oncolytic viral therapy regarding the definition of viral dosing functions relating to tumor size and disease burden. The volume of a tumor roughly scales as a function of tumor diameter raised to the third power ($V=\frac{\pi }{6}d^{3}$ for spherical tumors); typical dosing levels for oncolytic viruses do not reflect this. To illustrate the point, we show in Figure 4 the recommended upper limit of T-VEC injection volume (from Imlygic package insert) and calculated tumor volume as a function of tumor diameter. Of note, the literature on pharmacokinetic modeling of oncolytic virus effects within living systems appears to operate well using volumetric descriptions of tumors [63,64,65]; this raises the question of why clinical dosing of oncolytic viruses is not similarly volume-defined.

#### 4.3.4. What Is the Benefit of Injecting Primary Melanoma Lesions?

The question of which lesions should be injected in any given patient also remains unresolved. Data continues to accumulate, suggesting that not all types of injectable lesions equally elicit robust anti-tumor immunity [66]. The current recommended practice for T-VEC administration is to inject any new lesions first, followed by prioritization of remaining lesions by size, beginning with the largest lesion. This approach is perhaps reasonable if viewed through the perspective that new metastatic lesions and large lesions represent the greatest opportunities to induce anti-tumor immunity via T-VEC; however, a competing and yet entirely theoretical approach would be to prioritize the primary lesion (if not previously resected), given robust data demonstrating that primary lesions generally have a more immunostimulatory microenvironment compared to other sites. Compared to primary melanoma lesions, lymph node metastases have been demonstrated to have higher levels of indoleamine 2,3-dioxygenase (IDO), interleukin (IL)-10, regulatory T cells, and tolerogenic dendritic cells, which have the combined effect of creating an immunotolerant environment [67]. Primary melanomas have also been described to have a higher prevalence of lymphoid aggregates compared to metastatic lesions, independently correlating with overall survival on multivariate analysis [68]. These findings align with the well-described phenomenon of histologic regression noted in 9–58% of primary cutaneous melanomas [69,70], much higher than the 0.23% observed in metastatic lesions [71]. Histologic regression has been associated with favorable clinical outcomes, presumably through immune-mediated mechanisms [71,72,73,74]. A reasonable hypothesis based on these data is that oncolytic virus administration to primary melanoma lesions may have heightened potential over other types of lesions to elicit systemic anti-tumor immune responses. To date, this hypothesis remains untested. Of note, the phase II trial of T-VEC in the neoadjuvant setting allowed for injection of primary lesions; however, it remains unclear how many patients actually received injection to the primary lesion, and no analysis correlating patient outcomes with injected site type is presented [51]. A retrospective look at those data may potentially be informative, as will be any data coming out of NIVEC and KEYMAKER-U02 neoadjuvant trials [52,54].

#### 4.3.5. What Is the Benefit of Injecting Visceral Lesions?

The question of whether visceral metastases should be prioritized as injectable lesions is also becoming more important. With T-VEC, the data from the OPTiM phase III trial showed an ORR of 15% for non-injected visceral lesions [34]. The data from IGNYTE for RP1 are much stronger, with responses observed in 85.7% of injected visceral lesions and 65.4% of non-injected visceral lesions [75]. This expands the potential clinical utility of oncolytic virus therapy, as many patients with advanced disease have at least one visceral lesion. At present, oncolytic virus therapy is not capable of safely and effectively addressing osseous or central nervous system lesions.

#### 4.3.6. In What Other Melanoma Clinical Scenarios Might OVs Be Useful?

Much of the clinical interest in T-VEC and other OVs has focused on advanced disease. The neoadjuvant setting has a phase I pilot study is currently recruiting for use of RP1 in primary melanomas at high risk of lymph node metastases [76]. This represents a promising setting where oncolytic virus therapy may offer additional benefit. Another clinical scenario in which oncolytic viruses may potentially offer benefit in a new and emerging population is in high-risk stage II melanoma with regional lymph node failure while on adjuvant immune therapy. This remains to be explored.

#### 4.3.7. When Is Most Appropriate Time to Measure Efficacy Signals?

An additional unresolved question in the field of melanoma oncolytic virus clinical trials regards the optimal timepoint to measure efficacy signals. It is worth noting that the median time to response in the OPTiM trial with T-VEC was 4.1 months, and the median time to complete response was 8.6 months [34]. Slow responses to oncolytic virus therapy may therefore confound clinical trial designs looking at near-term efficacy endpoints and may confuse clinicians accustomed to changing therapy based on near-term response assessments. This delay in achieving clinical response to oncolytic viruses is notably similar to the literature describing delayed clinical responses to PD-1/PD-L1 immune checkpoint blockade in patients with melanoma and non-small cell lung cancer, with a clear benefit of continuing therapy even beyond clinical progression [77,78,79,80].

#### 4.3.8. Will Biomarkers Become Useful to Guide Patient Selection?

Multiple biomarkers have been proposed to predict responsiveness to OV therapy. Examples of proposed biomarkers include Nectin-1 expression, mutations in interferon (IFN)γ and Janus Kinase-Signal Transducer and Activator of Transcription (JAK-STAT), and peripheral blood immune cell composition [81,82,83]. None of these biomarkers have been validated prospectively, and additional barriers to translation include tumor heterogeneity and lack of standardized assays for clinical deployment [84,85,86]. Beyond this, the utility of a predictive biomarker for OV responsiveness in melanoma is debatable, as patients generally have few remaining treatment options, thus the results of biomarker analysis may not alter clinical management. The clinical utility of biomarkers for OV responsiveness in melanoma may potentially improve in proportion to the future expansion of alternative options for subsequent therapy.

## 5. Conclusions

Oncolytic virotherapy has significantly advanced from the initial empirical observations of viral infections associated with remission in hematologic malignancies to precision-engineered HSV-derived constructs designed to produce tumor-selective lysis and potent immune priming. Clinical experience to date, most notably with T-VEC given its FDA approval in 2015, and encouraging preliminary results from next-generation agents such as RP1, RP2, OH2, and OrienX010, which are still in clinical trials, demonstrates that intratumoral OV therapy can control local disease and elicit durable systemic anti-tumor immunity in subsets of patients with advanced melanoma. The clinical impact of OVs in advanced melanoma remains constrained by overall poor response rates, underwhelming activity in non-injected visceral lesions, and limited scope of the current T-VEC approval. The role of OVs in the neoadjuvant setting remains unclear in the era of preoperative anti-PD-1 therapy. Additional clinical scenarios that could benefit from OVs are envisioned but remain to be explored.

Multiple, interdependent translational barriers must be overcome to move oncolytic virotherapy from a niche intralesional tool to a broadly useful melanoma modality. Chief among these is defining robust synergistic OV and ICI combination regimens. Randomized, mechanism-driven studies with embedded immune correlative endpoints are needed to determine optimal combinations, sequencing, and injection site locations that produce durable systemic anti-tumor immunity. Biomarker work must move from correlative to prospective studies using validated assays. Future studies should additionally reconsider the definition of “injectable lesion” from both a technical feasibility and a biological standpoint, seeking to expand the relevance of OVs to additional clinical contexts in melanoma. Dosing functions for OVs should be reconsidered to more closely reflect tumor volume, and endpoints for future trials should reflect the delayed nature of responses to both OVs and ICIs. A key objective within the field remains the identification of specific, high-value clinical indications that can generate clear, regulatory-acceptable evidence. Regulatory uncertainty regarding the viability of pathways toward drug approval based off of single-arm trial data currently hinders clinical translation.

In the near term, clinicians should view OVs as a modality with particular value for patients with injectable disease or limited options after ICI failure. Over the longer term, iterative improvements in vector design, dosing paradigms, identification of maximally synergistic OV and ICI combinations, and biomarker-driven patient selection have the potential to expand the utility of oncolytic virotherapy in melanoma care.

## Figures and Tables

**Table 1:** Summary of HSV-1 variant oncolytic viruses that have been tested in clinical trials prior to T-VEC. Gene nomenclature is reflective of RefSeq assembly GCF_000859985.2. For information on any abbreviations used in this table, please refer to the list of abbreviations provided elsewhere in this manuscript.

Virus	Key Modifications	Clinical Outcome
G207	Insertional mutation of UL39 gene encoding ICP6 involved in evasion of host immune response.	Phase I trials in pediatric and adult high-grade gliomas showing modest improved OS compared to historical controls. Additional early phase trials in rhabdomyosarcoma, ovarian cancer, and colorectal cancer, with some hint of efficacy.
	Deletion of both copies of RL1 (γ34.5) gene encoding ICP34.5 involved in evasion of host cellular stress response.	
NV1020	Deletion of single copy of RL1 (γ34.5) gene encoding ICP34.5 involved in evasion of host cellular stress response.	Phase I/II trial of hepatic artery infusion in patients with liver-dominant metastatic colorectal cancer showing stabilization of liver lesions and modest radiosensitization.
	Deletion of both copies of UL56 gene encoding UL56 involved in viral spread and evasion of host immune response.	
	Deletion of UL24 gene required for efficient viral replication	
HSV1716	Spontaneous deletion of single copy of RL1 (γ34.5) gene encoding ICP34.5 involved in evasion of host cellular stress response.	Pilot study in metastatic melanoma showing some partial responses. Additional phase I/II trial in pleural mesothelioma showing DCR 50% but no objective responses. Early phase trials in high-grade gliomas showing possible OS benefit compared to historical controls.
HF10	Spontaneous deletion of genomic region containing UL43 (viral replication and assembly), UL49.5 (viral egress), UL55 (viral entry), and UL56 (capsid assembly), and US11 (γ134.5; evasion of host antiviral defense)	Phase II trial with Ipilimumab in cutaneous melanoma showing improved response rates compared to historical controls. Additional early phase trials in breast, pancreatic, and head and neck cancer, with variable modest efficacy.
T-VEC	Deletion of both copies of RL1 (γ34.5) gene encoding ICP34.5 involved in evasion of host cellular stress response.	Multiple phase II/III trials in melanoma. FDA approved in 2015 based on data from phase III OPTiM trial. Phase II trial data in cutaneous squamous cell carcinoma showing high response rates. Additional early phase trials in Merkel cell carcinoma and other solid tumors.
	Deletion of US12 gene encoding ICP47 involved in suppression of antigen presentation via MHC class I molecules.	

**Table 2:** Summary of notable phase II/III clinical trials of oncolytic viruses in advanced melanoma. Results shown only include pre-specified analyses from intention-to-treat population. No subgroup analyses are included. For information on any abbreviations used in this table, please refer to the list of abbreviations provided elsewhere in this manuscript.

**Trial Identifier**	OPTiM (NCT00769704)	MASTERKEY-265 (NCT02263508)	NCT01740297	IGNYTE (NCT03767348)
**Design**	Randomized, open-label phase III	Randomized, double-blinded, placebo-controlled phase III	Randomized, open-label phase II	Single-arm phase II
**Population**	Unresectable stage III-IV melanoma	Unresectable stage IIIB-IVM1c melanoma, prior BRAF-directed therapy allowed	Unresectable stage IIIB-IV melanoma with ≤1 line of prior therapy	Unresectable stage IIIB-IV melanoma with prior progression on anti-PD-1
**Arms**	T-VEC vs. GM-CSF	T-VEC + pembrolizumab vs. pembrolizumab	T-VEC + ipilimumab vs. ipilimumab	RP1 + nivolumab
**Injectable Lesions**	Cutaneous, subcutaneous, or nodal lesions ≥ 10 mm	Accessible visceral or soft tissue ≥ 10 mm, or nodal lesions ≥ 15 mm on short axis	Cutaneous, subcutaneous, or nodal melanoma ≥ 5 mm in longest diameter	Superficial or deep lesions ≥ 1 cm in longest diameter
**Primary Endpoint(s)**	DRR	PFS and OS (co-primary)	ORR	ORR
**Key Results**	DRR 16.3% vs. 2.1% (unadjusted OR, 8.9; 95% CI, 2.7 to 29.2; *p* < 0.001) ORR 26.4% (95% CI, 21.4 to 31.5) vs. 5.7% (95% CI, 1.9 to 9.5) (*p* < 0.001 [not prespecified]) Median OS 23.3 vs. 18.9 months (HR, 0.79; 95% CI, 0.62 to 1.00; *p* = 0.051)	Median PFS: 14.3 vs. 8.5 months (HR, 0.86; 95% CI, 0.71 to 1.04; *p* = 0.13) Median OS: NE vs. 49.2 months (HR of 0.96; 95% CI, 0.76 to 1.22; *p* = 0.74) ORR: 48.6% (95% CI, 43.3 to 53.8) vs. 41.3% (95% CI, 36.1 to 45.5) CRR: 17.9% (95% CI, 13.9 to 22.0) vs. 11.6% (95% CI, 8.2 to 14.9)	ORR: 35.7% vs. 16.0% (unadjusted OR of 2.9 (95% CI 1.5 to 5.7; *p* = 0.003) Median PFS: 13.5 vs. 6.4 months (HR 0.78; 95% CI 0.55 to 1.09; descriptive *p* = 0.14) Median OS: 84.9 vs. 50.1 months (HR 0.83; 95% CI 0.56 to 1.24, descriptive *p* = 0.37)	ORR: 32.9% (95% CI, 25.2% to 41.3%) DOR: 33.7 (95% CI, 14.1 to not reached) months CRR: 15% (95% CI, 9.5% to 22.0%) PFS: 3.6 (95% CI, 2.0–5.0) months 2-year OS: 63.3% (95% CI, 53.6% to 71.5%)
**≥Gr3 Safety Events**	11.3% vs. 4.7%	20.7% vs. 15.7%	46.3% vs. 43.2%	12.9%

**Table 3:** Summary of notable neoadjuvant phase II/III clinical trials of oncolytic viruses in melanoma. Results shown only include pre-specified analyses from intention-to-treat population. No subgroup analyses are included. For information on any abbreviations used in this table, please refer to the list of abbreviations provided elsewhere in this manuscript.

**Trial Identifier**	NCT02211131	NIVEC (NCT04330430)
**Design**	Randomized, open-label phase II	Single-arm open-label phase II
**Population**	Resectable stage IIIB-IVM1a melanoma	Resectable stage IIIB-IVA melanoma, no prior therapy
**Arms**	Neoadjuvant T-VEC vs. up-front surgery	Neoadjuvant T-VEC + nivolumab
**Injectable Lesions**	Cutaneous, subcutaneous, or nodal lesions ≥ 10 mm	Measurable lesion of ≥10 mm
**Primary Endpoint(s)**	RFS	Pathologic response rate
**Key Results**	2-year RFS: 29.5% vs. 16.5% (overall unstratified HR = 0.75, 80% CI = 0.58 to 0.96) 2-year LRFS: 36.5% vs. 27.5% (overall unstratified HR 0.83, 80% CI = 0.64 to 1.08) 2-year RRFS: 39.2% vs. 25.4% (overall unstratified HR 0.77, 80% CI = 0.59 to 1.01) 2-year DMFS: 33.7% vs. 19.5% (overall unstratified HR 0.74, 80% CI = 0.58 to 0.96) 2-year OS: 88.9% vs. 77.4% (overall unstratified HR 0.49, 80% CI = 0.30 to 0.79)	Pathologic response rate: 74% (MPR 65%) 1-year EFS: 75%
**≥Gr3 Safety Events**	5.5% in neoadjuvant arm	8%

## Data Availability

No new data were created or analyzed in this study.

## Abbreviations

The following abbreviations are used in this manuscript:

ATP	Adenosine triphosphate
CD	Cluster of differentiation
CI	Confidence interval
CR	Complete response
CRR	Complete response rate
CTLA-4	Cytotoxic T-lymphocyte associated protein-4
DAMP	Damage-associated molecular pattern
DCR	Disease-control rate
DMFS	Distant metastasis-free survival
DNA	Deoxyribonucleic acid
DRR	Durable response rate
EFS	Event-free survival
FDA	Food and drug administration
GALV-GP R	Gibbon ape leukemia virus fusogenic glycoprotein
GM-CSF	Granulocyte–monocyte colony-stimulating factor
HMGB1	High mobility group B1
HR	Hazard ratio
HSV	Herpes simplex virus
ICI	Immune checkpoint inhibitor
IDO	Indoleamine 2,3-dioxygenase
IL	Interleukin
LRFS	Local recurrence-free survival
MHC	Major histocompatibility complex
MPR	Major pathologic response
NCBI	National center for biotechnology information
NE	Not evaluable
OR	Odds ratio
ORF	Open reading frame
ORR	Overall response rate
OS	Overall survival
PD-1	Programmed cell death protein 1
PD-L1	Programmed cell death 1 ligand 1
PFS	Progression-free survival
PR	Partial response
RefSeq	NCBI reference sequence database
RFS	Recurrence-free survival
RP1	Replimune-1 (Vusolimogene oderparepvec)
RP2	Replimune-2
RRFS	Regional recurrence-free survival
SD	Stable disease
STING	Stimulator of interferon genes
TRAE	Treatment-related adverse event
T-VEC	Talimogene laherparepvec

## References

[1] Dock G. (1904). The Influence of Complicating Diseases Upon Leukaemia. Am. J. Med. Sci..

[2] Pelner L. (1958). Effects of Concurrent Infections and Their Toxins on the Course of Leukemia. Acta Med. Scand. Suppl..

[3] Sinkovics J., Horvath J. (1993). New Developments in the Virus Therapy of Cancer: A Historical Review. Intervirology.

[4] Bierman H.R., Crile D.M., Dod K.S., Kelly K.H., Petrakis N.I., White L.P., Shimkin M.B. (1953). Remissions in Leukemia of Childhood Following Acute Infectious Disease. Staphylococcus and Streptococcus, Varicella, and Feline Panleukopenias. Cancer.

[5] Hoster H.A., Zanes R.P., Von Haam E. (1949). Studies in Hodgkin’s Syndrome; the Association of Viral Hepatitis and Hodgkin’s Disease; a Preliminary Report. Cancer Res..

[6] Southam C.M., Moore A.E. (1952). Clinical Studies of Viruses as Antineoplastic Agents, with Particular Reference to Egypt 101 Virus. Cancer.

[7] Georgiades J., Zielinski T., Cicholska A., Jordan E. (1959). Research on the Oncolytic Effect of APC Viruses in Cancer of the Cervix Uteri; Preliminary Report. Biul. Inst. Med. Morsk. Gdan..

[8] Wheelock E.F., Dingle J.H. (1964). Observations on the Repeated Administration of Viruses to a Patient with Acute Leukemia. N. Engl. J. Med..

[9] Lawler S.E., Speranza M.C., Cho C.F., Chiocca E.A. (2017). Oncolytic Viruses in Cancer Treatment: A Review. JAMA Oncol..

[10] Watanabe D., Goshima F. (2018). Oncolytic Virotherapy by HSV. Adv. Exp. Med. Biol..

[11] Nemunaitis J., Senzer N., Sarmiento S., Zhang Y.A., Arzaga R., Sands B., Maples P., Tong A.W. (2007). A Phase I Trial of Intravenous Infusion of ONYX-015 and Enbrel in Solid Tumor Patients. Cancer Gene Ther..

[12] Khuri F.R., Nemunaitis J., Ganly I., Arseneau J., Tannock I.F., Romel L., Gore M., Ironside J., MacDougall R.H., Heise C. (2000). A Controlled Trial of Intratumoral ONYX-015, a Selectively-Replicating Adenovirus, in Combination with Cisplatin and 5-Fluorouracil in Patients with Recurrent Head and Neck Cancer. Nat. Med..

[13] Galanis E., Okuno S.H., Nascimento A.G., Lewis B.D., Lee R.A., Oliveira A.M., Sloan J.A., Atherton P., Edmonson J.H., Erlichman C. (2005). Phase I-II Trial of ONYX-015 in Combination with MAP Chemotherapy in Patients with Advanced Sarcomas. Gene Ther..

[14] McGeoch D.J. (1987). The Genome of Herpes Simplex Virus: Structure, Replication and Evolution. J. Cell Sci..

[15] Martuza R.L., Malick A., Markert J.M., Ruffner K.L., Coen D.M. (1991). Experimental Therapy of Human Glioma by Means of a Genetically Engineered Virus Mutant. Science.

[16] Markert J.M., Malick A., Coen D.M., Martuza R.L. (1993). Reduction and Elimination of Encephalitis in an Experimental Glioma Therapy Model with Attenuated Herpes Simplex Mutants That Retain Susceptibility to Acyclovir. Neurosurgery.

[17] Mineta T., Rabkin S.D., Yazaki T., Hunter W.D., Martuza R.L. (1995). Attenuated Multi-Mutated Herpes Simplex Virus-1 for the Treatment of Malignant Gliomas. Nat. Med..

[18] Todo T., Feigenbaum F., Rabkin S.D., Lakeman F., Newsome J.T., Johnson P.A., Mitchell E., Belliveau D., Ostrove J.M., Martuza R.L. (2000). Viral Shedding and Biodistribution of G207, a Multimutated, Conditionally Replicating Herpes Simplex Virus Type 1, after Intracerebral Inoculation in Aotus. Mol. Ther..

[19] Sundaresan P., Hunter W.D., Martuza R.L., Rabkin S.D. (2000). Attenuated, Replication-Competent Herpes Simplex Virus Type 1 Mutant G207: Safety Evaluation in Mice. J. Virol..

[20] Markert J.M., Liechty P.G., Wang W., Gaston S., Braz E., Karrasch M., Nabors L.B., Markiewicz M., Lakeman A.D., Palmer C.A. (2009). Phase Ib Trial of Mutant Herpes Simplex Virus G207 Inoculated Pre-and Post-Tumor Resection for Recurrent GBM. Mol. Ther..

[21] Cozzi P.J., Burke P.B., Bhargav A., Heston W.D.W., Huryk B., Scardino P.T., Fong Y. (2002). Oncolytic Viral Gene Therapy for Prostate Cancer Using Two Attenuated, Replication-Competent, Genetically Engineered Herpes Simplex Viruses. Prostate.

[22] Ebright M.I., Zager J.S., Malhotra S., Delman K.A., Weigel T.L., Rusch V.W., Fong Y. (2002). Replication-Competent Herpes Virus NV1020 as Direct Treatment of Pleural Cancer in a Rat Model. J. Thorac. Cardiovasc. Surg..

[23] Cozzi P.J., Malhotra S., McAuliffe P., Kooby D.A., Federoff H.J., Huryk B., Johnson P., Scardino P.T., Heston W.D., Fong Y. (2001). Intravesical Oncolytic Viral Therapy Using Attenuated, Replication-Competent, Herpes Simplex Viruses G207 and Nv1020 Is Effective in the Treatment of Bladder Cancer in an Orthotopic Syngeneic Model. FASEB J..

[24] Geevarghese S.K., Geller D.A., De Haan H.A., Hörer M., Knoll A.E., Mescheder A., Nemunaitis J., Reid T.R., Sze D.Y., Tanabe K.K. (2010). Phase I/II Study of Oncolytic Herpes Simplex Virus NV1020 in Patients with Extensively Pretreated Refractory Colorectal Cancer Metastatic to the Liver. Hum. Gene Ther..

[25] Kemeny N., Brown K., Covey A., Kim T., Bhargava A., Brody L., Guilfoyle B., Haag N.P., Karrasch M., Glasschroeder B. (2006). Phase I, Open-Label, Dose-Escalating Study of a Genetically Engineered Herpes Simplex Virus, NV1020, in Subjects with Metastatic Colorectal Carcinoma to the Liver. Hum. Gene Ther..

[26] Sze D.Y., Iagaru A.H., Gambhir S.S., De Haan H.A., Reid T.R. (2012). Response to Intra-Arterial Oncolytic Virotherapy with the Herpes Virus NV1020 Evaluated by [^18^F]Fluorodeoxyglucose Positron Emission Tomography and Computed Tomography. Hum. Gene Ther..

[27] MacKie R.M., Stewart B., Brown S.M. (2001). Intralesional Injection of Herpes Simplex Virus 1716 in Metastatic Melanoma. Lancet.

[28] Andtbacka R.H., Ross M.I., Agarwala S.S., Taylor M.H., Vetto J.T., Neves R.I., Daud A., Khong H.T., Ungerleider R.S., Tanaka M. (2017). Final Results of a Phase II Multicenter Trial of HF10, a Replication-Competent HSV-1 Oncolytic Virus, and Ipilimumab Combination Treatment in Patients with Stage IIIB-IV Unresectable or Metastatic Melanoma. J. Clin. Oncol..

[29] Liu B.L., Robinson M., Han Z.Q., Branston R.H., English C., Reay P., McGrath Y., Thomas S.K., Thornton M., Bullock P. (2003). ICP34.5 Deleted Herpes Simplex Virus with Enhanced Oncolytic, Immune Stimulating, and Anti-Tumour Properties. Gene Ther..

[30] Moesta A.K., Cooke K., Piasecki J., Mitchell P., Rottman J.B., Fitzgerald K., Zhan J., Yang B., Le T., Belmontes B. (2017). Local Delivery of OncoVEXmGM-CSF Generates Systemic Antitumor Immune Responses Enhanced by Cytotoxic T-Lymphocyte–Associated Protein Blockade. Clin. Cancer Res..

[31] Estrada J., Zhan J., Mitchell P., Werner J., Beltran P.J., Devoss J., Qing J., Cooke K.S. (2023). OncoVEX MGM-CSF Expands Tumor Antigen-Specific CD8+ T-Cell Response in Preclinical Models. J. Immunother. Cancer.

[32] Bommareddy P.K., Zloza A., Rabkin S.D., Kaufman H.L. (2019). Oncolytic Virus Immunotherapy Induces Immunogenic Cell Death and Overcomes STING Deficiency in Melanoma. Oncoimmunology.

[33] Gartrell R.D., Blake Z., Rizk E.M., Perez-Lorenzo R., Weisberg S.P., Simoes I., Esancy C., Fu Y., Davari D.R., Barker L. (2022). Combination Immunotherapy Including OncoVEXmGMCSF Creates a Favorable Tumor Immune Micro-Environment in Transgenic BRAF Murine Melanoma. Cancer Immunol. Immunother..

[34] Andtbacka R.H., Collichio F., Harrington K.J., Middleton M.R., Downey G., Öhrling K., Kaufman H.L. (2019). Final Analyses of OPTiM: A Randomized Phase III Trial of Talimogene Laherparepvec versus Granulocyte-Macrophage Colony-Stimulating Factor in Unresectable Stage III-IV Melanoma. J. Immunother. Cancer.

[35] Andtbacka R.H., Kaufman H.L., Collichio F., Amatruda T., Senzer N., Chesney J., Delman K.A., Spitler L.E., Puzanov I., Agarwala S.S. (2015). Talimogene Laherparepvec Improves Durable Response Rate in Patients with Advanced Melanoma. J. Clin. Oncol..

[36] DailyMed—IMLYGIC—Talimogene Laherparepvec Injection, Suspension. https://dailymed.nlm.nih.gov/dailymed/drugInfo.cfm?setid=64ffb680-ea8c-42fc-9649-9e8c0eb77ddb&audience=consumer.

[37] Long G., Dummer R., Johnson D., Michielin O., Martin-Algarra S., Treichel S., Chan E., Diede S., Ribas A. (2020). 429 Long-Term Analysis of MASTERKEY-265 Phase 1b Trial of Talimogene Laherparepvec (T-VEC) plus Pembrolizumab in Patients with Unresectable Stage IIIB-IVM1c Melanoma. J. Immunother. Cancer.

[38] Chesney J.A., Ribas A., Long G.V., Kirkwood J.M., Dummer R., Puzanov I., Hoeller C., Gajewski T.F., Gutzmer R., Rutkowski P. (2023). Randomized, Double-Blind, Placebo-Controlled, Global Phase III Trial of Talimogene Laherparepvec Combined with Pembrolizumab for Advanced Melanoma. J. Clin. Oncol..

[39] Chesney J.A., Puzanov I., Collichio F.A., Singh P., Milhem M.M., Glaspy J., Hamid O., Ross M., Friedlander P., Garbe C. (2023). Talimogene Laherparepvec in Combination with Ipilimumab versus Ipilimumab Alone for Advanced Melanoma: 5-Year Final Analysis of a Multicenter, Randomized, Open-Label, Phase II Trial. J. Immunother. Cancer.

[40] Wong M.K., Milhem M.M., Sacco J.J., Michels J., In G.K., Couselo E.M., Schadendorf D., Beasley G.M., Niu J., Chmielowski B. (2025). RP1 Combined with Nivolumab in Advanced Anti-PD-1-Failed Melanoma (IGNYTE). J. Clin. Oncol..

[41] Ribas A., Kirkwood J.M., Flaherty K.T. (2018). Anti-PD-1 Antibody Treatment for Melanoma. Lancet Oncol..

[42] Luke J.J., Kong G., Gullo G., Robert C. (2024). A Randomized, Controlled, Multicenter, Phase 3 Study of Vusolimogene Oderparepvec (VO) Combined with Nivolumab vs Treatment of Physician’s Choice in Patients with Advanced Melanoma That Has Progressed on Anti–PD-1 and Anti–CTLA-4 Therapy (IGNYTE-3). J. Clin. Oncol..

[43] Wang X., Tian H., Chi Z., Si L., Sheng X., Hu H., Gu X., Li S., Li C., Lian B. (2025). Oncolytic Virus OH2 Extends Survival in Patients with PD-1 Pretreated Melanoma: Phase Ia/Ib Trial Results and Biomarker Insights. J. Immunother. Cancer.

[44] Cui C.L., Wang X., Lian B., Ji Q., Zhou L., Chi Z., Si L., Sheng X., Kong Y., Yu J. (2022). OrienX010, an Oncolytic Virus, in Patients with Unresectable Stage IIIC-IV Melanoma: A Phase Ib Study. J. Immunother. Cancer.

[45] Guo J., Cui C., Wang X., Lian B., Yin S., Cong Y., Chi Z., Si L., Sheng X., Tang B. (2021). A Phase 1b Clinical Trial of Anti-PD-1 Ab (Toripalimab) plus Intralesional Injection of OrienX010 in Stage Melanoma with Liver Metastases. J. Clin. Oncol..

[46] Zarezadeh Mehrabadi A., Tat M., Ghorbani Alvanegh A., Roozbahani F., Esmaeili Gouvarchin Ghaleh H. (2024). Revolutionizing Cancer Treatment: The Power of Bi- and Tri-Specific T-Cell Engagers in Oncolytic Virotherapy. Front. Immunol..

[47] Harrington K.J., Sacco J.J., Olsson-Brown A.C., Chan T.Y., Nenclares P., Leslie I., Saleem I., Bommareddy P., Ahlers C.M., Coffin R.S. (2022). A Phase 1 Trial of RP2, a First-in-Class, Enhanced Potency Oncolytic HSV Expressing an Anti-CTLA-4 Antibody as a Single Agent and Combined with Nivolumab in Patients with Advanced Solid Tumors. J. Clin. Oncol..

[48] Sacco J.J., Harrington K.J., Olsson-Brown A., Chan T.Y., Nenclares P., Leslie I., Bommareddy P., Kalbasi A., Xie B., Mishal M. (2024). Safety, Efficacy, and Biomarker Results from an Open-Label, Multicenter, Phase 1 Study of RP2 Alone or Combined with Nivolumab in a Cohort of Patients with Uveal Melanoma. J. Clin. Oncol..

[49] Sacco J.J., Moser J.C., Betof Warner A., Sullivan R.J., Davar D., Shoushtari A.N., Carvajal R.D., Patel S.P., Joshua A.M., Hassel J.C. (2025). A Randomized, Phase 2/3 Clinical Trial Investigating RP2 plus Nivolumab vs Ipilimumab plus Nivolumab in Immune Checkpoint Inhibitor-Naïve Patients with Metastatic Uveal Melanoma. J. Clin. Oncol..

[50] Study Details|NCT05938296|OHSV2-PD-L1/CD3-BsAb Administered via Intratumoral Injection|ClinicalTrials.gov. NCT05938296.

[51] Dummer R., Gyorki D.E., Hyngstrom J.R., Ning M., Lawrence T., Ross M.I. (2023). Final 5-Year Follow-Up Results Evaluating Neoadjuvant Talimogene Laherparepvec Plus Surgery in Advanced Melanoma: A Randomized Clinical Trial. JAMA Oncol..

[52] Zijlker L.P., van Houdt W.J., Stahlie E.H., Franke V., Rohaan M.W., Delatzakis A., Zuur C., Klop W.M., van de Wiel B.A., Kuijpers A. (2023). Neoadjuvant T-VEC + Nivolumab Combination Therapy for Resectable Early Metastatic (Stage IIIB/C/D-IV M1a) Melanoma with Injectable Disease: NIVEC Trial. J. Clin. Oncol..

[53] Liu J., Wang X., Li Z., Gao S., Mao L., Dai J., Li C., Cui C., Chi Z., Sheng X. (2024). Neoadjuvant Oncolytic Virus Orienx010 and Toripalimab in Resectable Acral Melanoma: A Phase Ib Trial. Signal Transduct. Target. Ther..

[54] Dummer R., Robert C., Scolyer R.A., Taube J.M., Tetzlaff M.T., Menzies A.M., Hill A., Grob J.J., Portnoy D.C., Lebbe C. (2025). Neoadjuvant Anti-PD-1 Alone or in Combination with Anti-TIGIT or an Oncolytic Virus in Resectable Stage IIIB–D Melanoma: A Phase 1/2 Trial. Nat. Med..

[55] Sacco J.J., Harrington K.J., Olsson-Brown A., Chan T.Y., Nenclares P., Leslie I., Bommareddy P., Xie B., Wolff J., Middleton M.R. (2023). Preliminary Safety and Efficacy Results from an Open-Label, Multicenter, Phase 1 Study of RP2 as a Single Agent and in Combination with Nivolumab in a Cohort of Patients with Uveal Melanoma. J. Clin. Oncol..

[56] FDA Issues CRL for RP1 in Advanced Melanoma. https://www.targetedonc.com/view/fda-issues-crl-for-rp1-in-advanced-melanoma.

[57] Beekman K.E., Parker L.M., DePalo D.K., Elleson K.M., Sarnaik A.A., Tsai K.Y., Withycombe B.M., Zager J.S. (2023). Four Cases of Disseminated Herpes Simplex Virus Following Talimogene Laherparepvec Injections for Unresectable Metastatic Melanoma. JAAD Case Rep..

[58] Study Details|NCT04349436|A Phase 1B/2 Study of RP1 in Solid Organ Transplant Patients with Advanced Cutaneous Malignancies|ClinicalTrials.gov. NCT04349436.

[59] Migden M.R., Chai-Ho W., Daniels G.A., Medina T., Wise-Draper T.M., Kheterpal M., Tang J.C., Ibrahim S.F., Bolotin D., Verschraegen C. (2024). Abstract CT003: Initial Results from an Open-Label Phase 1b/2 Study of RP1 Oncolytic Immunotherapy in Solid Organ Transplant Recipients with Advanced Cutaneous Malignancies (ARTACUS). Cancer Res..

[60] Andtbacka R.H., Amatruda T., Nemunaitis J., Zager J.S., Walker J., Chesney J.A., Liu K., Hsu C.P., Pickett C.A., Mehnert J.M. (2019). Biodistribution, Shedding, and Transmissibility of the Oncolytic Virus Talimogene Laherparepvec in Patients with Melanoma. eBioMedicine.

[61] Harrington K.J., Kong A., Mach N., Chesney J.A., Fernandez B.C., Rischin D., Cohen E.E.W., Radcliffe H.S., Gumuscu B., Cheng J. (2020). Talimogene Laherparepvec and Pembrolizumab in Recurrent or Metastatic Squamous Cell Carcinoma of the Head and Neck (MASTERKEY-232): A Multicenter, Phase 1b Study. Clin. Cancer Res..

[62] Study Details|NCT04599062|TVEC and Preop Radiation for Sarcoma (8 mL Dose)|ClinicalTrials.gov. NCT04599062.

[63] Parra-Guillen Z.P., Sancho-Araiz A., Mayawala K., Zalba S., Garrido M.J., de Alwis D., Troconiz I.F., Freshwater T. (2023). Assessment of Clinical Response to V937 Oncolytic Virus After Intravenous or Intratumoral Administration Using Physiologically-Based Modeling. Clin. Pharmacol. Ther..

[64] Jenner A.L., Kim P.S., Frascoli F. (2019). Oncolytic Virotherapy for Tumours Following a Gompertz Growth Law. J. Theor. Biol..

[65] Wu J.T., Kirn D.H., Wein L.M. (2004). Analysis of a Three-Way Race between Tumor Growth, a Replication-Competent Virus and an Immune Response. Bull. Math. Biol..

[66] Dougherty K.E., Peery G.B., Agala C.B., Pham V.P., Gwynn M.E., Meyers M.O., Stitzenberg K.B., Sorah J.D., Long P.K., Jones C.P. (2025). Anatomic Location Predicts Response Rates: Real World Outcomes with Over 1,100 Cycles of Talimogene Laherparepvec (TVEC) Description: A Retrospective Review of a Prospectively Maintained Database. Ann. Surg..

[67] Polak M.E., Borthwick N.J., Gabriel F.G., Johnson P., Higgins B., Hurren J., McCormick D., Jager M.J., Cree I.A. (2007). Mechanisms of Local Immunosuppression in Cutaneous Melanoma. Br. J. Cancer.

[68] Karapetyan L., Li A., Vargas De Stefano D., Abushukair H.M., Al-Bzour A.N., Knight A., Layding C., Wang H., Xu J., Yao J. (2024). Differences in the Pathological, Transcriptomic, and Prognostic Implications of Lymphoid Structures between Primary and Metastatic Cutaneous Melanomas. J. Immunother. Cancer.

[69] Blessing K., McLaren K.M. (1992). Histological Regression in Primary Cutaneous Melanoma: Recognition, Prevalence and Significance. Histopathology.

[70] Måsbäck A., Westerdahl J., Ingvar C., Olsson H., Jonsson N. (1997). Cutaneous Malignant Melanoma in Southern Sweden 1965, 1975, and 1985. Cancer.

[71] Kalialis L.V., Drzewiecki K.T., Klyver H. (2009). Spontaneous Regression of Metastases from Melanoma: Review of the Literature. Melanoma Res..

[72] El Sharouni M.A., Aivazian K., Witkamp A.J., Sigurdsson V., Van Gils C.H., Scolyer R.A., Thompson J.F., Van Diest P.J., Lo S.N. (2021). Association of Histologic Regression with a Favorable Outcome in Patients with Stage 1 and Stage 2 Cutaneous Melanoma. JAMA Dermatol..

[73] Subramanian S., Han G., Olson N., Leong S.P., Kashani-Sabet M., White R.L., Zager J.S., Sondak V.K., Messina J.L., Pockaj B. (2021). Regression Is Significantly Associated with Outcomes for Patients with Melanoma. Surgery.

[74] Cervinkova M., Kucerova P., Cizkova J. (2017). Spontaneous Regression of Malignant Melanoma—Is It Based on the Interplay between Host Immune System and Melanoma Antigens?. Anticancer Drugs.

[75] Wong M.K., Sacco J.J., Robert C., Michels J., Bowles T.L., In G.K., Tsai K.K., Lebbe C., Gaudy-Marqueste C., Muñoz Couselo E. (2024). Efficacy and Safety of RP1 Combined with Nivolumab in Patients with Anti–PD-1–Failed Melanoma from the IGNYTE Clinical Trial. J. Clin. Oncol..

[76] Study Details|NCT06216938|RP1 in Primary Melanoma to Reduce the Risk of Sentinel Lymph Node Metastasis|ClinicalTrials.gov. NCT06216938.

[77] Kazandjian D., Keegan P., Suzman D.L., Pazdur R., Blumenthal G.M. (2017). Characterization of Outcomes in Patients with Metastatic Non-Small Cell Lung Cancer Treated with Programmed Cell Death Protein 1 Inhibitors Past RECIST Version 1.1–Defined Disease Progression in Clinical Trials. Semin. Oncol..

[78] Pourmir I., Elaidi R., Maaradji Z., De Saint Basile H., Ung M., Ismaili M., Fournier L., Rance B., Gibault L., Ben Dhiab R. (2023). Longitudinal Study of Advanced Non-Small Cell Lung Cancer with Initial Durable Clinical Benefit to Immunotherapy: Strategies for Anti-PD-1/PD-L1 Continuation beyond Progression. Cancers.

[79] Chen C., Xiong X., Cheng Y., Gen H., Zhu W., Zhang F., Zhu C., Han S., Liu X. (2023). Expanding the Applications of Immune Checkpoint Inhibitors in Advanced Lung Cancer beyond Disease Progression. Front. Immunol..

[80] Beaver J.A., Hazarika M., Mulkey F., Mushti S., Chen H., He K., Sridhara R., Goldberg K.B., Chuk M.K., Chi D.C. (2018). Patients with Melanoma Treated with an Anti-PD-1 Antibody beyond RECIST Progression: A US Food and Drug Administration Pooled Analysis. Lancet Oncol..

[81] Schwertner B., Lindner G., Toledo Stauner C., Klapproth E., Magnus C., Rohrhofer A., Gross S., Schuler-Thurner B., Öttl V., Feichtgruber N. (2021). Nectin-1 Expression Correlates with the Susceptibility of Malignant Melanoma to Oncolytic Herpes Simplex Virus In Vitro and In Vivo. Cancers.

[82] Nguyen T.T., Ramsay L.A., Ahanfeshar-Adams M., Lajoie M., Schadendorf D., Alain T., Watson I.R. (2021). Mutations in the IFNγ-JAK-STAT Pathway Causing Resistance to Immune Checkpoint Inhibitors in Melanoma Increase Sensitivity to Oncolytic Virus Treatment. Clin. Cancer Res..

[83] Kudling T.V., Bychkov D., Clubb J.H., Pakola S.A., Arias V., Jirovec E., van der Heijden M., Ojala N., Quixabeira D.C., Haybout L. (2025). Single-Cell Profiling of Peripheral Blood Mononuclear Cells from Patients Treated with Oncolytic Adenovirus TILT-123 Reveals Baseline Immune Status as a Predictor of Therapy Outcomes. Cancer Gene Ther..

[84] Nassief G., Anaeme A., Moussa K., Chen D., Ansstas G. (2024). Where Are We Now with Oncolytic Viruses in Melanoma and Nonmelanoma Skin Malignancies?. Pharmaceuticals.

[85] Wang J.W., Feng Q., Liu J.H., Xun J.J. (2025). Opportunities, Challenges, and Future Perspectives of Oncolytic Virus Therapy for Malignant Melanoma. Front. Immunol..

[86] Kaufman H.L. (2021). Can Biomarkers Guide Oncolytic Virus Immunotherapy?. Clin. Cancer Res..

